# Prolonged acute kidney injury exacerbates lung inflammation at 7 days post‐acute kidney injury

**DOI:** 10.14814/phy2.12084

**Published:** 2014-07-22

**Authors:** Ana Andres‐Hernando, Christopher Altmann, Rhea Bhargava, Kayo Okamura, Jasna Bacalja, Brandi Hunter, Nilesh Ahuja, Danielle Soranno, Sarah Faubel

**Affiliations:** 1 Department of Medicine, University of Colorado Denver, Aurora, Colorado

**Keywords:** Fluid overload, ischemic acute kidney injury, lung inflammation

## Abstract

Patients with acute kidney injury (AKI) have increased mortality; data suggest that the duration, not just severity, of AKI predicts increased mortality. Animal models suggest that AKI is a multisystem disease that deleteriously affects the lungs, heart, brain, intestine, and liver; notably, these effects have only been examined within 48 h, and longer term effects are unknown. In this study, we examined the longer term systemic effects of AKI, with a focus on lung injury. Mice were studied 7 days after an episode of ischemic AKI (22 min of renal pedicle clamping and then reperfusion) and numerous derangements were present including (1) lung inflammation; (2) increased serum proinflammatory cytokines; (3) liver injury; and (4) increased muscle catabolism. Since fluid overload may cause respiratory complications post‐AKI and fluid management is a critical component of post‐AKI care, we investigated various fluid administration strategies in the development of lung inflammation post‐AKI. Four different fluid strategies were tested – 100, 500, 1000, or 2000 *μ*L of saline administered subcutaneously daily for 7 days. Interestingly, at 7 days post‐AKI, the 1000 and 2000 *μ*L fluid groups had *less severe *AKI and less severe lung inflammation versus the 100 and 500 *μ*L groups. In summary, our data demonstrate that appropriate fluid management after an episode of ischemic AKI led to both (1) faster recovery of kidney function and (2) significantly reduced lung inflammation, consistent with the notion that interventions to shorten AKI duration have the potential to reduce complications and improve patient outcomes.

## Abstract (precis)

e12084

Our data demonstrate that prolonged AKI is associated with lung inflammation up to 7 days post‐AKI consistent with the notion that duration of AKI is important in the adverse outcomes associated with AKI.

## Introduction

Acute kidney injury (AKI) injury occurs in 20% of hospital admissions (Uchino et al. 2006) and 30–50% of admissions to the intensive care unit (ICU; Star 1998) and is associated with increased mortality. Increased mortality may be due to deleterious systemic effects of AKI. Indeed, AKI is associated with numerous complications such as respiratory failure, prolonged mechanical ventilation, and heart failure. Animal studies confirm these clinical observations as well as provide mechanistic insights – AKI causes lung injury (Kramer et al. 1999; Hassoun et al. 2007), cardiac injury (Kelly 2003), liver injury (Yildirim et al. 2003; Kim et al. 2011), brain injury (Liu et al. 2008), and intestinal injury (Park et al. 2012). Notably, however, these complications have only been studied within 48 h and longer term effects of an episode of experimental AKI are unknown.

In the present study, we sought to examine the longer term systemic effects of AKI, with a focus on lung injury. Although we (Hoke et al. 2007; Klein et al. 2008) and others (Heidland et al. 1984; Kramer et al. 1999; Rabb et al. 2003; Deng et al. 2004; Nath et al. 2005; Kim do et al. 2006; Hassoun et al. 2007; Grigoryev et al. 2008) have shown that AKI in animals leads to lung inflammation similar to that observed in animal models of acute lung injury (ALI; Kim do et al. 2006), it is still debated whether the respiratory complications of AKI are solely due to fluid overload, or whether there is indeed a direct effect of AKI on lung inflammation and injury. Indeed, fluid overload is an independent predictor of increased mortality in AKI (Payen et al. 2008; Goldstein 2012) that is thought to contribute to numerous adverse consequences in AKI (Butcher and Liu 2012), in addition to respiratory complications. Given the importance of fluid management and avoidance of fluid overload in AKI, we also studied the effect of various fluid dosing strategies on kidney function recovery and lung inflammation.

We found that an episode of ischemic AKI is associated with numerous systemic derangements 1‐week post‐AKI, including lung inflammation. Using various fluid dosing strategies, we were able to affect kidney function recovery at 7 days, without affecting kidney injury severity at 24 h. We found that faster resolution of AKI was associated with less lung inflammation 7 days post‐AKI. Emerging data suggest that the duration of AKI is a key factor that predicts adverse outcomes in AKI (Coca et al. 2010; Palevsky et al. 2013). In the present study, appropriate fluid management post‐AKI led to both faster recovery of kidney function and significantly reduced lung inflammation, consistent with the notion that interventions to shorten AKI duration have the potential to reduce complications and improve patient outcomes.

## Methods

### Animals

Eight‐ to 10‐week‐old C57BL/6 mice (Jackson Laboratories, Bar Harbor, ME) that weighed 20–25 g were used. Mice were maintained on a standard diet, and water was freely available. All experiments were conducted with adherence to the National Institutes of Health Guide for the Care and Use of Laboratory Animals. The animal protocol was approved by the Animal Care and Use Committee of the University of Colorado, Denver.

### Surgical protocol for sham operation and ischemic AKI

Surgical procedures were performed on two groups of mice: (1) sham operation and (2) ischemic AKI. For all procedures, mice were anesthetized with intraperitoneal avertin (2,2,2‐tribromoethanol; Sigma Aldrich, Milwaukee, WI), a midline incision was made, and the renal pedicles were identified. In the ischemic AKI group, both renal pedicles were clamped for 22 min. After clamp removal, kidneys were observed for restoration of blood flow by the return to original color. The abdomen was closed in one layer. Sham surgery consisted of the same procedure except that clamps were not applied.

### Collection and preparation of serum samples

At sacrifice, blood was obtained via cardiac puncture. To ensure uniformity, all samples were processed identically. Blood was allowed to clot at room temperature for 2 h, and then centrifuged at 3000 *g* for 10 min. Serum was collected and centrifuged a second time at 3000 *g* for 1 min to ensure elimination of red blood cells. Samples with notable hemolysis were discarded.

### Serum cytokine measurement

Serum cytokines were analyzed on a Mouse ProInflammatory 7‐Plex Ultra‐Sensitive Kit (cat # K15012C; MesoScale Discovery, Gaithersburg, MD),

### Serum endotoxin concentration

Serum endotoxin levels were determined using a kinetic, turbidimetric Limulus Amebocyte Lysate (LAL; PYROGENT‐5000 catalog #N383; Lonza, Walkesville, MD) endotoxin detection assay, as per the manufacturer's directions. To confirm adequate technique a positive control was studied – serum endotoxin level was determined 4 h after IP injection of 0.01 mg endotoxin and was 11,988 endotoxin units (*n* = 1).

### Blood cultures

Peripheral blood was obtained via heart puncture under sterile conditions and 0.5 mL was transferred into a sterile tube containing 0.5 mL of BacT/ALERT SA blood culture medium (bioMérieux, Inc., Durham, NC). Two hundred microliter aliquots of this dilution were spread onto duplicate LB agar plates, and the plates were incubated at 37°C. The bacterial colonies on the plates were counted. To confirm adequate technique, a positive control was studied – peritoneal swabs were obtained 4 h after the sepsis model of cecal ligation and puncture, and showed numerous bacterial colonies within 24 h.

### Assessment of kidney function and kidney injury

Blood urea nitrogen and serum creatinine were measured using a VetAce autoanalyzer (Alfa Wassermann, West Caldwell, NJ). Urine NGAL was measured by ELISA (R&D Systems, Minneapolis, MN) following the manufacturer's instructions (detection level 8.8 pg/mL).

Formaldehyde (4%)‐fixed and paraffin‐embedded kidneys were sectioned at 4 *μ*m and stained with periodic acid–Schiff (PAS) by standard methods. All histological examinations were performed by the renal pathologist in a blinded fashion. Histological changes due to ATN score were evaluated in the outer stripe of the outer medulla on PAS‐stained tissue and were quantified by counting the percent of tubules that displayed cell necrosis, loss of brush border, cast formation, and tubule dilatation as follows: 0, none; 1, <10%; 2, 11–25%; 3, 26–45%; 4, 46–75%; and 5, >76%. At least 10 fields (200×) were reviewed for each slide.

### Lung CXCL1

CXCL1 is a neutrophil chemokine, analogous to human IL‐8. Lung CXCL1 was performed on whole lung homogenates by ELISA (R&D Systems) and corrected for protein (detection level 2.0 pg/mL). Tissue was prepared as described previously (Klein et al. 2008).

### Lung neutrophil activity (myeloperoxidase activity)

Lung myeloperoxidase (MPO) activity is a biochemical marker of neutrophils and neutrophil activation. One fourth of lung was homogenized in 1 mL of cold hexadecyltrimethlylammonium bromide buffer (50 mmol/L KPO_4_ and 0.5% hexadecyltrimethylammonium bromide [pH 6.0]), sonicated on ice for 10 sec, and centrifuged at 14,000 *g* for 30 min at 4°C. Twenty microliter of supernatant was transferred into a 96‐well plate, and 200 *μ*L of 37°C *O*‐dianisidine hydrochloride solution (16.7 mg *O*‐dianisidine, 100 mL: 90% water and 10% 50 mmol/L KPO4 buffer + 0.0005% H_2_O_2_) was added immediately before the optical density was read at 450 nm and again 30 sec later (Benchmark microplate reader; BioRad, Hercules, CA).

### Lung neutrophil content (flow cytometry)

To confirm and quantify lung neutrophil accumulation, flow cytometry for lung neutrophils was performed. Perfused right lower lobe of lungs were minced and incubated for 30 min at 37°C in RPMI media containing 2 mg/mL collagenase B, then mashed through a 70‐*μ*m nylon cell strainer (BD Falcon, Franklin Lakes, NJ). Cells were incubated with Fc Block (CD16/32; eBiosciences, San Diego, CA) before staining with specific antibodies. The following rat anti‐mouse antibodies were used for surface staining – F4/80‐PerCP‐Cy5, CD11b‐Pacific Blue and CD11c‐PECy7 (eBiosciences), CD45‐V500 and Ly6G‐APC‐Cy7 (BD Pharmingen, San Jose, CA).

Multicolor multiparameter flow cytometry was performed using a FACSCanto II instrument (BD Biosciences, San Jose, CA) compensated with single fluorochromes and analyzed using Diva^™^ software (BD Biosciences).

### Statistical analysis

Data were compared with Student's *t*‐test for two experimental groups, or with ANOVA when comparing more than two experimental groups (for Fig. 6, Dunnett's multiple comparison test was used for the AKI group using the 2000 *μ*L group as the control; for Fig. 8, Tukey's multiple comparison test was used). Results were expressed as mean ± SEM. All statistical analysis was performed with GraphPad Prism 5.0 statistical software (La Jolla, CA).

## Results

### Effects of AKI at 7 days

To evaluate the longer term systemic effects of AKI, male C57Bl/6 mice were subjected to ischemic AKI or sham operation and the following were determined 7 days postprocedure: (1) kidney injury and kidney function, (2) lung inflammation, (3) serum cytokines, (4) liver injury, (5) weight loss, (6) serum glutamate, and (7) hematocrit.

This ischemic AKI model is characterized by a dramatic rise in serum creatinine to approximately 3.0 mg/dL (from a baseline of 0.2–0.3 mg/dL) and an increase in blood urea nitrogen (BUN) to approximately 100–150 mg/dL by 24 h postischemic reperfusion (Hoke et al. 2007; Klein et al. 2008; Andres‐Hernando et al. 2011), kidney function – as judged by BUN and creatinine – is typically normal by 72 h postischemic reperfusion.

#### Kidney injury and kidney function

**Figure 1.**
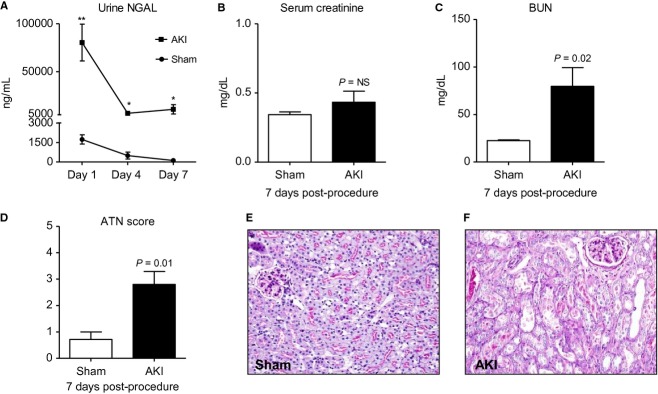
Kidney injury and kidney function 7 days after acute kidney injury (AKI). To assess kidney injury, (A) urine NGAL was determined on day 1, 4, and 7 after sham operation (Sham) and AKI. To assess kidney function, (B) serum creatinine and (C) blood urea nitrogen were determined 7 days postprocedure. To further quantify kidney injury, kidney histology was examined and (D) acute tubular necrosis scores were determined. Representative histology 7 days post (E) sham operation and (F) AKI. *n* = 7–9. Mice were administered 500 *μ*L of saline daily. **P < 0.005, *P < 0.05 versus Sham.

Urine NGAL (a marker of tubular injury) was dramatically increased on day 1 post‐AKI suggesting that mice receiving AKI surgery developed tubular injury and AKI. Seven days post‐AKI, BUN, but not serum creatinine was significantly increased. To assess kidney injury, kidney histology was examined and acute tubular necrosis (ATN) scores were significantly increased 7 days post‐AKI. Thus, even though serum creatinine was back to baseline and similar to sham, kidney injury and dysfunction were still present 7 days post‐AKI as judged by urine NGAL, BUN, and kidney histology (ATN score; Fig. 1).

#### Lung inflammation and serum cytokines 7 days post‐AKI

**Figure 2.**
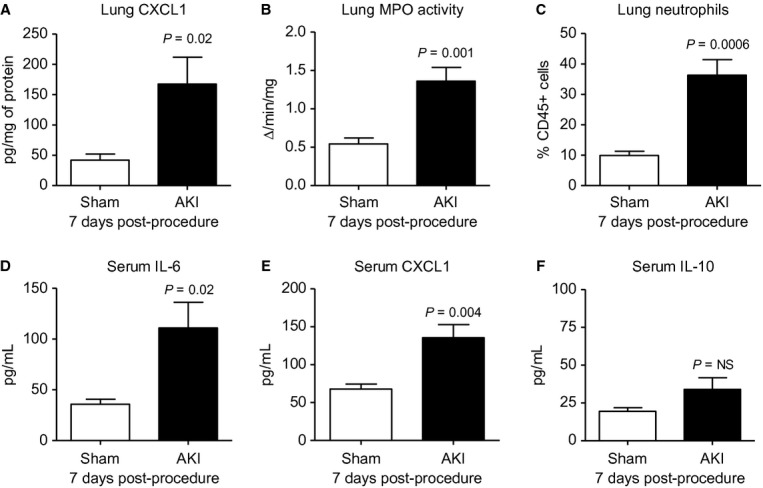
Lung inflammation and serum cytokines 7 days after acute kidney injury (AKI). One week after sham operation (Sham) or AKI lung inflammation and serum cytokines were determined. Lung inflammation was worse as judged by (A) lung CXCL1 (a neutrophil chemokine), (B) lung myeloperoxidase (MPO) activity (a marker of neutrophil activation), and (C) lung neutrophil content (by flow cytometry). Serum (D) IL‐6 and (E) CXCL1 were increased. Serum (F) IL‐10 was not increased. *n* = 7–10. Mice were administered 500 *μ*L of saline daily.

We and others have demonstrated that serum cytokines and lung inflammation increased 4 and 24 h after AKI (Hoke et al. 2007); however, neither of these endpoints have been examined beyond 48 h in animal models. As shown in Figure 2, lung inflammation and the serum proinflammatory cytokines IL‐6 and CXCL1 were increased 7 days post‐AKI, but the anti‐inflammatory cytokine IL‐10 was not. Increased serum IL‐6 and IL‐8 (analogous to CXCL1) are well known to be increased in patients with AKI and predict prolonged mechanical ventilation (Liu et al. 2009) and increased mortality (Simmons et al. 2004). Serum IL‐1*β*, TNF‐*α*, IFN‐*γ*, and IL‐12, were not increased 7 days post‐AKI (data not shown). In summary, these data demonstrate that lung and systemic inflammation are present 7 days post‐AKI, even in the presence of a normal serum creatinine.

#### Liver and spleen injury and inflammation 7 days post‐AKI

**Figure 3.**
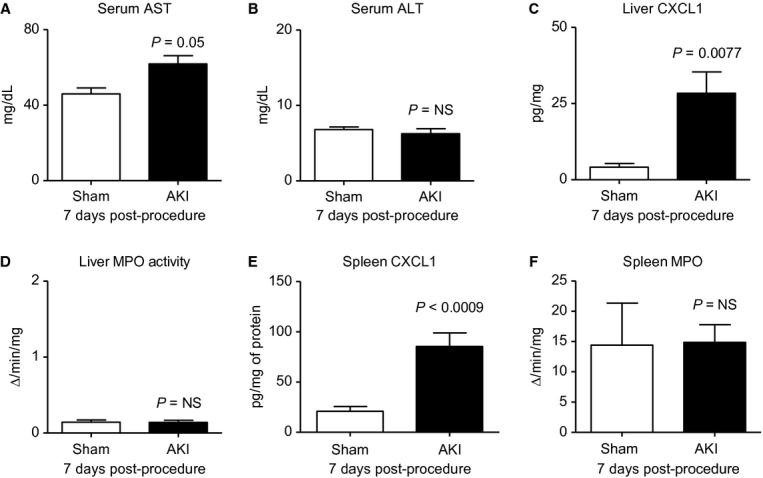
Liver and spleen inflammation 7 days after acute kidney injury (AKI). One week after sham operation (Sham) or AKI, liver function (serum AST, ALT) and liver inflammation (liver CXCL1, liver MPO activity), and splenic inflammation (spleen CXCL1, spleen MPO activity) were determined. MPO: myeloperoxidase activity, a biochemical marker of neutrophils; *n* = 7–10. Mice were administered 500 *μ*L of saline daily.

Studies have demonstrated that cytokine‐mediated liver injury and inflammation occur 5 and 24 h after AKI (Park et al. 2011), although longer term assessment of liver injury has not been examined. As shown in Figure 3, features of liver injury were still present 7 days post‐AKI as judged by increased serum AST and increased liver CXCL1; serum ALT and liver MPO activity were not increased. Similarly, increased cytokine production has been observed in the spleen within 24 h post‐AKI, yet longer term assessment of splenic inflammation has not been examined. As shown in Figure 3, splenic CXCL1 was increased 7 days post‐AKI, but splenic MPO activity was not increased. In summary, these data demonstrate that a marker of inflammation and stress (CXCL1) is increased in the liver and spleen up to 1 week post‐AKI.

#### Weight loss, serum glutamate, and hematocrit 7 days post‐AKI

**Figure 4.**
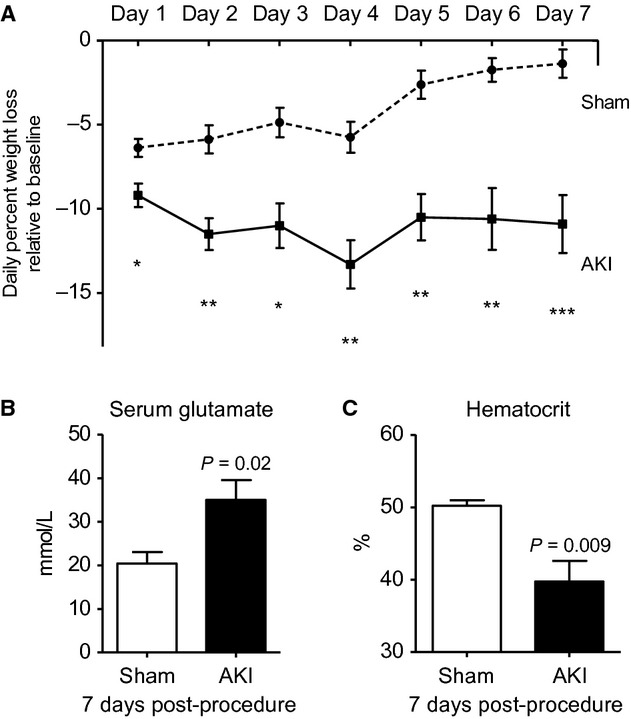
Weight loss, serum glutamate, and hematocrit 7 days after acute kidney injury (AKI). (A) Weight loss relative to baseline weight was assessed every day after sham operation (Sham) or AKI for 7 days. One week after Sham or AKI, (B) serum glutamate and (C) hematocrit was assessed. *n* = 7–10.

Weight loss is a well‐known marker of overall health in mice, therefore, weight loss was determined daily, and as shown in Figure 4A, mice lost weight after both sham operation and AKI beginning on day 1, although weight loss was greater on all days in mice with AKI. By day 7, sham‐operated mice had returned to their normal weight, while mice with AKI still were approximately 10% below baseline weight.

Because of the significant weight loss in the AKI group, we questioned whether mice with AKI might be hypercatabolic. Serum glutamate is a marker of muscle breakdown in hypercatabolic patients in several settings, including critical illness; therefore, we measured serum glutamate on day 7 postprocedure. As shown in Figure 4B, serum glutamate was significantly increased in mice with AKI demonstrating that muscle breakdown and a hypercatabolic state are present.

To further investigate the cause of the weight loss after AKI, we determined hematocrit. Hematocrit is a well‐established marker of hydration status in animals, with higher hematocrit being associated with volume depletion and fluid loss (Dennen et al. 2010). Thus, if weight loss after AKI were due to fluid loss, then the hematocrit would be increased. Contrary to expectations, hematocrit was significantly *decreased* in mice with AKI. Since blood loss is not a feature of the ischemic AKI model, we reasoned that the decrease in hematocrit was due to hemodilution and *fluid overload* as mice had received 500 *μ*L of fluid daily to maintain hydration.

#### Blood cultures and serum endotoxin levels

Since AKI is associated with respiratory complications and fluid overload is thought to contribute, we considered the role of fluid overload in mediating the lung inflammation we observed 7 day post‐AKI. A leading hypothesis regarding the deleterious effects fluid overload is that fluid overload may lead to gut edema and subsequent bacteria and/or endotoxin release thus causing sepsis and/or the systemic inflammatory response syndrome (SIRS; Butcher and Liu 2012); since SIRS is associated with lung inflammation, we considered the possibility that bacteremia or endotoxemia might be present 7 days post‐AKI. Therefore, blood cultures and serum endotoxin activity were determined 7 days postprocedure.

**Figure 5.**
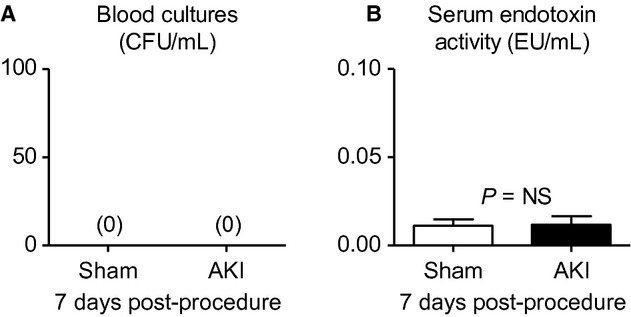
Blood cultures and serum endotoxin levels 7 days after acute kidney injury (AKI). (A) Blood cultures and (B) serum endotoxin levels were determined 7 days postsham operation (sham) and AKI in mice administered 500 *μ*L 0.9% NS daily. (*n* = 4).

As shown in Figure 5, blood cultures assessed 7 days after sham or AKI showed no growth after 72 h (*n* = 4); similarly, serum endotoxin levels (endotoxin units/mL) assessed 7 days after sham or AKI were similar and were 0.0112 ± 0.0003 in sham and 0.0118 ± 0.005 (*P* = NS, *n* = 5) indicating virtually no serum endotoxin activity in either group. Thus, neither bacteremia nor increased endotoxin activity are responsible for the increase in systemic inflammation observed post‐AKI.

#### Effect of various fluid administration strategies on the development of lung inflammation 7 days post‐AKI

We next questioned whether fluid overload might directly contribute to the increased lung inflammation observed 7 days post‐AKI. Fluid overload is well known to contribute to respiratory complications in patients with AKI, although it is generally assumed that fluid overload in the setting of AKI results in cardiogenic pulmonary edema, and not pulmonary inflammation.

Therefore, to specifically address whether fluid administration leading to fluid overload was contributing to lung inflammation in the setting of AKI, several different fluid administration strategies were examined. Specifically, we tested the effect of 100, 500, 1000, and 2000 *μ*L of sterile saline administered subcutaneously immediately postprocedure and daily thereafter in mice after sham operation and AKI (the last dose of fluid was administered 2 h prior to sacrifice); we also tested the effect of no fluid administration and fluid administration to maintain preprocedure body weight.

#### Mortality

No fluid administration after AKI resulted in >50% mortality within 48 h. The administration of fluid to maintain pre‐AKI body weight resulted in >50% mortality within 24 h. Therefore, no further experiments were performed with these fluid administration strategies. In mice with AKI, mortality was 45% with 100 *μ*L daily, 12% with 500 *μ*L daily, 0% with 1000 *μ*L daily, and 0% with 2000 *μ*L daily. No mortality was observed in sham‐operated mice with any of the fluid administration.

#### Effect of 100 *μ*L daily saline

**Figure 6.**
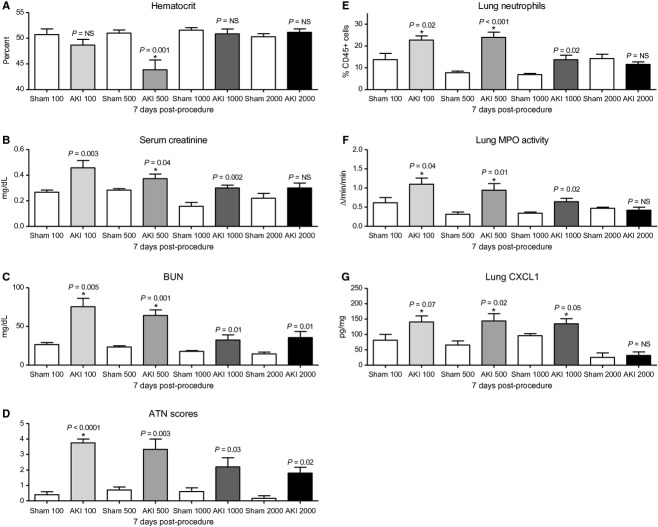
The effect of four different fluid administration strategies on hematocrit, kidney function, and lung inflammation 7 days after acute kidney injury (AKI). Mice received 100, 500, 1000, or 2000 *μ*L of fluid beginning immediately postprocedure and then daily for 7 days after sham operation (Sham 100, Sham 500, Sham 1000, or Sham 2000, respectively) or AKI (AKI 100, AKI 500, AKI 1000, or AKI 2000, respectively) and (A) hematocrit, (B) serum creatinine, (C) blood urea nitrogen, (D) acute tubular necrosis scores, (E) lung neutrophils by flow cytometry, (F) lung myeloperoxidase activity and (G) lung CXCL1 were determined on day 7 postprocedure. *P* values above columns are for comparisons between AKI and Sham of the same fluid strategy; *P < 0.05 versus AKI 2000.

As expected, administration of 100 *μ*L of saline did not result in a decrement in hematocrit 7 days post‐AKI, suggesting that hemodilution and volume overload were not present; however, lung inflammation as judged by percent lung neutrophils, lung MPO activity, and lung CXCL1 accumulation were increased versus sham. Notably, significant renal dysfunction was still observed as judged by an increase in both serum creatinine and BUN (Fig. 6). The increase in serum creatinine and BUN suggesting worse kidney dysfunction was confirmed by the significant increase in histologic kidney injury as judged by increased ATN score 7 days post‐AKI.

#### Effect of 500 *μ*L of daily saline

As above (Figs. 1–4), 500 *μ*L of saline resulted in a decrease in hematocrit, suggesting hemodilution and volume overload, which was associated with increased lung inflammation (increased lung neutrophils, lung MPO activity, and lung CXCL1); in this experiment, both serum creatinine and BUN were still increased at 7 days versus sham (Fig. 6).

#### Effect of 1000 and 2000 *μ*L of daily saline

Unexpectedly, administration of either 1000 or 2000 *μ*L of saline daily did not result in volume overload at 7 days post‐AKI and hematocrit was no different than sham. Although an exact record of urine output was not obtained, we observed that mice administered 1000 or 2000 *μ*L of fluid daily would urinate nearly the entire volume of fluid almost immediately after administration beginning on day 3 or 4 post‐AKI, while mice that received 100 or 500 *μ*L of saline had minimal urine output throughout the experiment. Confirming these clinical observations that suggested faster recovery of kidney function in the 1000 and 2000 *μ*L groups, serum creatinine and BUN were normal 7 days post‐AKI in these groups (Fig. 6). Notably, lung inflammation was minimally increased in the 1000 *μ*L group, and was absent in the 2000 *μ*L group (Fig. 6).

Remarkably, these data suggest that kidney function recovery can be significantly modulated by various fluid administration strategies and that delayed recovery of kidney function, with or without fluid overload, is associated with increased lung inflammation that is present 7 days post‐AKI.

#### Relationship between lung neutrophil content and BUN

**Figure 7.**
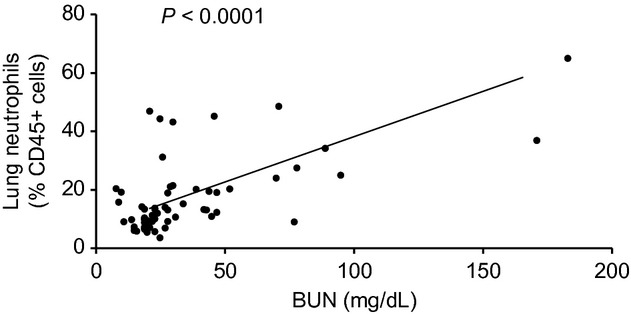
Relationship between lung neutrophil content and blood urea nitrogen (BUN). The correlation between lung neutrophil percent and BUN was assessed for all acute kidney injury experiments, and was highly significant (*P* < 0.0001) with higher BUN levels being correlated with higher lung neutrophil content (*n* = 61).

To further examine the relationship between kidney function recovery and lung inflammation, BUN levels were correlated with lung neutrophil percentages (from *all* AKI experiments) and the correlation was highly significant (*P* < 0.0001) with higher levels of BUN correlating with higher levels of lung neutrophils (Fig. 7, *n* = 61).

#### Effect of various fluid administration strategies on the development of lung inflammation 24 h post‐AKI

**Figure 8.**
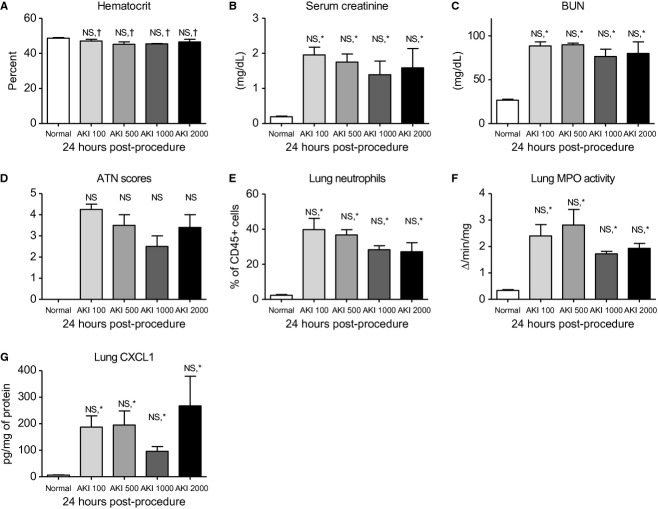
The effect of four different fluid administration strategies on hematocrit, kidney function and kidney injury, and lung inflammation 24 h after acute kidney injury (AKI). Mice received 100, 500, 1000, or 2000 of fluid beginning immediately postprocedure and then 22 h after AKI (AKI 100, AKI 500, AKI 1000, or AKI 2000, respectively). (A) Hematocrit, (B) serum creatinine, (C) blood urea nitrogen, (D) acute tubular necrosis scores, (E) lung neutrophils, (F) lung myeloperoxidase activity, and (G) lung CXCL1 were determined 24 h postprocedure. Normal mice (unmanipulated) were also studied. NS: not significant for AKI 100 versus AKI 500 versus AKI 1000 versus AKI 2000 with one way ANOVA and Tukey comparing all columns; †not significant versus normal; *P < 0.05 versus normal.

To confirm that the effect on lung inflammation was due to prolonged AKI, and not an immediate beneficial effect of increased fluid administration, we tested the effect of 100, 500, 1000, and 2000 *μ*L of sterile saline administered subcutaneously immediately postprocedure and at 22 h with sacrifice at 24 h postprocedure. As shown in Figure 8, no significant differences were observed among 100, 500, 1000, and 2000 *μ*L fluid administration at 24 h post‐AKI regarding hematocrit, serum creatinine, BUN, ATN scores, lung neutrophils, lung MPO activity, or lung CXCL1, thus confirming that the severity of AKI and lung inflammation were similar 24 h post‐AKI among the four group, yet the two groups with delayed recovery of AKI (100 and 500 *μ*L had increased lung inflammation at 7 days).

## Discussion

Acute kidney injury causes numerous complications and increases mortality. Clinical and experimental data suggest that AKI is a multisystem disease that is associated with respiratory complications, metabolic derangements, liver injury, brain dysfunction, and intestinal injury. Much attention has focused on the role of severity of AKI in predicting complications, poor outcomes, and increased mortality. Indeed, increased severity of AKI is associated with a corresponding increased risk of mortality, with more severe AKI being associated with a higher risk of death (Ricci et al. 2008). Data are accumulating that the *duration* of AKI may be an additional, if not more, important factor in predicting poor outcomes (Palevsky et al. 2013). In one study, duration of AKI was associated with increased risk of mortality and, after adjustment for duration of AKI, staging/severity of AKI was no longer predictive of adverse outcomes (Coca et al. 2010; Palevsky et al. 2013). Our data in the present study are consistent with this emerging concept and demonstrate that prolonged AKI is associated with significant lung inflammation at 7 days post‐AKI. Although delayed kidney function recovery might be expected to be associated with worse systemic effects, this association has not been previously examined or reported in experimental AKI. Thus, our data in the present study lend important insights into understanding the respiratory complications associated with AKI, as well as the effect of fluid therapy in ischemic AKI recovery.

Acute kidney injury is associated with numerous pulmonary complications that include respiratory failure (Metnitz et al. 2002; Waikar et al. 2007; Walcher et al. 2011), prolonged mechanical ventilation (Vieira et al. 2007; Liu et al. 2009; Parikh et al. 2011), and prolonged ventilator weaning (Vieira et al. 2007); and the development of respiratory complications increases mortality. Indeed, combined AKI and respiratory failure carries mortality rates between 60% and 80%. The fact that AKI confers an increased risk of respiratory complications has been recognized for over 100 years (Lange 1901; Rackow et al. 1978). Similarly, the role of fluid overload in respiratory complications post‐AKI has been debated for over 100 years, with some advocating that fluid overload is the sole reason for respiratory failure in AKI (Alwall et al. 1953) and other concluding that AKI causes severe lung injury in the form of the adult respiratory distress syndrome (ARDS; Bleyl et al. 1981; Faubel 2008). Central to the pathogenesis of ARDS is lung chemokine upregulation, neutrophil accumulation, and neutrophil activation (Ware and Matthay 2000). Our data in the present study demonstrate that lung chemokine upregulation, neutrophil accumulation, and neutrophil activation are dramatically increased 7 days post‐AKI with or without fluid overload. Thus, these data put to rest the notion that pulmonary complications post‐AKI are solely related to fluid overload.

Appropriate fluid management is a critical component of the care of patients with AKI, and optimizing fluid management in AKI may ultimately reduce complications and mortality in AKI (Faubel et al. 2012). A wealth of convincing evidence has demonstrated that fluid overload is associated with increased mortality in AKI and is associated with numerous deleterious systemic effects (Goldstein et al. 2005; Payen et al. 2008); thus, current trends have been to recommend a fluid conservative strategy in patients with AKI. Our data demonstrate that in ischemic AKI, a very conservative fluid strategy was detrimental and resulted in delayed recovery of kidney function and lung inflammation 7 days post‐AKI. We also demonstrate that recovery after ischemic AKI may be modulated by various fluid administration strategies identifying a simple model to further study the effects of delayed kidney function recovery on outcomes post‐AKI.

Our report has a number of limitations. (1) Blood pressure were not measured and it is possible that low blood pressure from inadequate fluid administration is the cause of the exacerbation of AKI in the mice receiving less fluid administration (i.e., the 100 and 500 *μ*L per day vs. the 1000 and 2000 *μ*L per day). Since inadequate fluid administration led to the prolongation of AKI, it is reasonable to assume that these mice were either prerenal or indeed had decreased blood pressure. Since low blood pressure alone is not known to cause lung inflammation, we believe that prolonged AKI (regardless of the cause) mediates the lung inflammation we observed 1 week post‐AKI. (2) Hematocrit is used to assess volume status and fluid overload without confirmatory measure. Despite this, data suggest that hematocrit may be a very reliable measure of hemodilution and hemoconcentration and is a well‐established marker of volume status in veterinary medicine. Since hematocrit is the percent (%) of red blood cell (RBC) volume relative to total blood volume, reduced plasma volume (dehydration) increases hematocrit, while increased plasma volume (fluid overload) *decreases* hematocrit. Indeed, our published data demonstrate that normal hematocrit is approximately 50% in mice which increases to 52–60% with volume depletion from diuretic administration (Metnitz et al. 2002). In the current study, hematocrit decreased to 40% with 500 *μ*L daily fluid administration (Fig. 1). Since normal plasma volume is approximately 500 *μ*L and our model of AKI causes decreased urine output, it is plausible that daily administration of 500 *μ*L of 0.9% not significant (NS) would exceed daily urine output and cause fluid overload. Reduced hematocrit could also be a marker of reduced RBCs, however, blood loss and anemia are not features of the AKI model as evident in the 24‐h post‐AKI data showing similar hematocrit to normal (Fig. 7A), and the 7‐day hematocrit data demonstrating normal hematocrit in the other three fluid administration strategy groups (Fig. 6A). It is implausible that blood loss is responsible for this change since hematocrits were similar among all the AKI groups 24 h postprocedure; it is also implausible that other factors such as a change in erythropoietin production would only be evident at 7 days in one of the four AKI groups (the 500 *μ*L group), especially since creatinine levels were similar in the 100 and 500 *μ*L group. Thus, we are confident that hematocrit is a reasonable surrogate for hemodilution and volume overload in the AKI model. (3) BUN and creatinine were the primary measurements of kidney dysfunction. It is possible that the reduction in BUN and serum creatinine 7 days post‐AKI represented better hydration in the 1000 and 2000 *μ*L groups and did not necessarily correlate with better kidney function as we have ascertained. Measurement of glomerular filtration rate (GFR) would be necessary to confirm that changes in BUN and creatinine correlated with changes in kidney function. It should be noted, however, that increasing ATN scores (representing histologic kidney injury) did correlate with higher levels of BUN and serum creatinine in our experiments.

In summary, we demonstrate for the first time that multiple systemic derangements are present up to 7 days after ischemic AKI. In particular, lung inflammation occurred in the absence of fluid overload and was significantly related to prolonged duration of AKI, but not initial severity of AKI. These data lend important insights into the pathogenesis of respiratory complications in patients with AKI and demonstrate that delayed recovery of kidney function impairs the resolution of lung inflammation post‐AKI.

## Conflict of Interest

None declared.
